# Dynamic observation and qualitative analysis of a psychological crisis hotline during the COVID-19 pandemic

**DOI:** 10.3389/frhs.2022.968025

**Published:** 2022-08-31

**Authors:** Mengyuan Ouyang, Shasha Song, Hui Ma, Hua Yang, Jing Leng, Ping Zhou, Changjun Teng, Hongxia Ou, Jijun Li, Na Liu, Ning Zhang

**Affiliations:** ^1^The Affiliated Nanjing Brain Hospital of Nanjing Medical University, Nanjing, China; ^2^Department of Medical Psychology, The Affiliated Brain Hospital of Nanjing Medical University, Nanjing, China

**Keywords:** COVID-19, psychological crisis hotline, dynamic observation, qualitative analysis, public

## Abstract

**Objective:**

The aim of this study was to analyze the chief complaints of psychological crisis hotlines during the coronavirus disease 2019 (COVID-19) pandemic in Jiangsu, China, and to summarize the psychological characteristics of the public during the different stages of COVID-19.

**Methods:**

The chief complaints of calls to the psychological crisis hotline from 27 January 2020 to 30 June 2020. A total of 578 calls were extracted and grouped using thematic analysis into categories. After statistical analysis, the monthly and three-period trends were observed dynamically to determine whether there were statistical differences in the proportion of specific chief complaints over the phases.

**Results:**

There were a total of 495 cases of psychological problems or physical discomfort, accounting for 85.64% of the total sample number of hotline calls related to the pandemic. The numbers of callers with anxiety, depression, obsessive-compulsive symptoms, illness anxiety, insomnia, and physical discomfort were 370 (64.01%), 103 (17.99%), 33 (5.71%), 36 (6.23%), 51 (8.82%), and 72 (12.46%), respectively, and 83 (14.36%) callers consulted other problems. The monthly main complaints showed a fluctuating trend, and each main complaint peaked at different stages. The main complaints during the three stages had distinct features, respectively, and the proportions of calls for the specific complaints differed statistically over the phases.

**Conclusion:**

Dynamic observation and qualitative analysis of psychological crisis hotline data might indicate dynamic changes and accordingly provide guidance for online crisis intervention when other public health crises occur.

## Introduction

Coronavirus disease 2019 (COVID-19) has been the focus of attention since December 2019. It has widely and rapidly spread in China and several other countries, causing an outbreak of acute infectious pneumonia. The increasing numbers of patients, suspected cases, and outbreak-affected provinces and countries have elicited public worry about becoming infected (1). Anxiety, depression, stress, and sleep problems were commonly reported among the public during the pandemic, and higher suicidal risk at population level was one of the most important concerns (2). In the face of COVID-19, which is highly contagious and requires quarantine measures, hotlines have become the most convenient and feasible first choice for psychological assistance. The National Health Commission immediately issued guidelines for emergency psychological crisis intervention for people affected by COVID-19 (3) and subsequently announced the establishment of a psychological crisis hotline (4). Online psychological crisis intervention for the public was opened in all provinces across China. The internet hospital gradually opened up more service resources to provide psychological counseling and clinical diagnosis and treatment for patients, their family members, and other people affected by the epidemic. The Nanjing Crisis Intervention Center is the first domestic professional institution for crisis intervention and suicide prevention established on 1 July 1991. The crisis intervention center has a psychological crisis intervention clinic and a psychological crisis intervention hotline. The Jiangsu Provincial Psychological Crisis Intervention Hotline was established in 2007, and the hotline was linked to the Nanjing Crisis Intervention Center. At the same time, since 2012, we can also get help by dialing Nanjing Health 12,320 hotline, realizing three-line integration. Subsequently, a 24-h psychological hotline was set up to provide professional psychological crisis intervention services to prevent psychological stress due to the epidemic. Individuals in different age groups were psychologically affected by different tendencies at different stages of the epidemic. Therefore, by analyzing the main complaints of the three psychological crisis hotlines during the COVID-19 epidemic, this study dynamically observed the public's psychological response under specific crisis situations. It will provide guidance for psychological crisis intervention hotline work in the future, particularly for similar public health issues.

## Methods

### Study setting

Starting from 27 January 2020, the hotline services have been provided from 8:00 to 18:00, and it was established for 24-h operation on 7 February 2020. Approximately 100 psychiatrists, psychological consultants, and psychotherapy professionals from the Jiangsu Psychological Crisis Center in Nanjing Brain Hospital affiliated with Nanjing Medical University participated in the hotline work as volunteers. At the outset, they received uniform or standardized training and supervision to record callers' complaints, as well as monthly group supervision.

### Inclusion and exclusion criteria

Only calls where the caller specifically referenced COVID-19 or the pandemic were included. Calls related to emotional problems, marital problems, children's education, family problems, psychological crisis, mental illness medication, etc., where COVID-19 was not discussed as a factor, were excluded. The following calls were also excluded: (1) the caller's main purpose was not seeking psychological services and (2) “null” calls (i.e., silence, harassing, or hoax calls). For repeat calls from the same caller, only the first call was included in the analysis.

### Qualitative analysis

Dynamic records of the psychological hotline for epidemic prevention were updated over 5 months from 27 January to 30 June 2020 and extracted from the Jiangsu Psychological Crisis Center in Nanjing Brain Hospital affiliated with Nanjing Medical University. Based on previous studies, the theme analysis was adopted to extract the key information of the main complaints from the call records to determine the content and meaning. Classification analysis was used to classify the callers into the following categories: (1) psychological problems such as anxiety, depression, obsessive-compulsive symptoms, illness anxiety, and insomnia; (2) physical discomfort; and (3) other problems, including pandemic-related quarantine, registration, drug procurement, local policy consultation, etc. Words with obvious emotional indications such as worry, nervousness, and fear in the call records were classified as anxiety; calls regarding fear of infection or suspicion of suffering from a certain disease or sequelae were classified as illness anxiety; emotional depression, moodiness, pessimism, feelings of unfairness, and suicidal thoughts were classified as depression; fear of getting dirty, repetitive handwashing and checking, and disinfection were classified as obsessive-compulsive symptoms; poor sleep and difficulty in falling asleep were classified as insomnia; self-reported symptoms such as low-grade fever, cough, and chest pain without any evidence of COVID-19 infection were classified as physical discomfort. Categories were not mutually exclusive. All categories we defined as appeared in each call.

### Dynamic observation

We divided the period from 27 January to 30 June into three phases according to the dynamic changes in the COVID-19 epidemic in China, namely, the peak period (27 January to– 29 February) when the majority of Chinese people were quarantined at home during the peak of COVID-19 infection; the prerelease period (1 March to 30 April) before quarantine release and resumption; and the resumption period (1 May to 30 June) when most workplaces and schools were gradually reopened. The statistical products and services solution (IBM SPSS 25.0) was used for quantitative analysis, such as calculating the proportion of each category to the total calls. The monthly and three-period trends were dynamically observed by plotting. Finally, the chi-square test was used to determine whether the proportions of calls for the specific complaints differed statistically over the phases.

## Results

A total of 4,319 psychological hotline calls were received from 27 January to 30 June 2020. A total of 1,180 call records were related to the epidemic, 602 repeated or harassing calls were excluded, and a total of 578 were finally included in the data analysis. Among them, 495 were about psychological problems or physical discomfort, and 83 were simply about consulting other problems.

### General information

Some information, including name, gender, age, education, and job, was missing because some callers were unwilling to provide private information. A total of 489 individuals disclosed their sex in this analysis sample, including 215 women (43.97%) and 274 men (56.03%). A total of 310 individuals provided specific ages, including 4 participants (1.29%) from 0 to 18 years old, 247 participants (79.68%) from 19 to 45 years old, 47 participants (15.16%) from 46 to 60years old, and 12 participants (3.87%) >60 years old. The results showed that the proportion of male callers was slightly higher than that of female callers, and the majority of callers were 19–45 years old. Specific medical history was provided in 139 cases (24.05%), mainly including mental disorders such as depression, bipolar disorder, anxiety disorder, schizophrenia, insomnia, and somatic diseases such as hypertension, diabetes, chronic pharyngitis, and Parkinson's disease. There were 141 callers (24.39%) with a medication history, of which 57 (9.86%) provided specific medications, mainly lorazepam, sertraline, escitalopram, olanzapine, and fluoxetine. Overall, there were a total of 495 callers that had psychological problems or physical discomfort, accounting for 85.64% of the total sample number of hotline calls related to the epidemic.

### Consultation content

The main results were as follows: (1) psychological problems: anxiety (*n* = 370, 64.01%), depression (*n* = 103, 17.99%), obsessive-compulsive symptoms (*n* = 33, 5.71%), illness anxiety (*n* = 36, 6.23%), and insomnia (*n* = 51, 8.82%); (2) physical discomfort (*n* = 72, 12.46%); and (3) other problems (*n* = 83, 14.36%).

### Multiple psychological problems

As shown in Table 1, some callers reported having both psychological problems. Anxiety was still the main complaint, and most callers were nervous and worried because of the fear of contacting or getting infected with the new coronavirus. A small number also reported three or more symptoms, most of which included anxiety and insomnia. Obsessive-compulsive symptoms are also common co-symptoms. Other symptoms were mainly physical discomfort, depression, and illness anxiety.

**Table 1:** Two kinds of symptoms.

**Complaint N**	**Illness anxiety**	**Anxiety**	**OC symptoms**	**Depression**	**Physical discomfort**
Anxiety	16	-	-	-	-
OC symptoms	0	29	-	-	-
Depression	3	38	14	-	-
Physical discomfort	5	66	11	6	-
Insomnia	0	41	3	8	9

OC, obsessive-compulsive.

### Dynamic changes in chief complaints

#### Monthly trend

**Figure 1:**
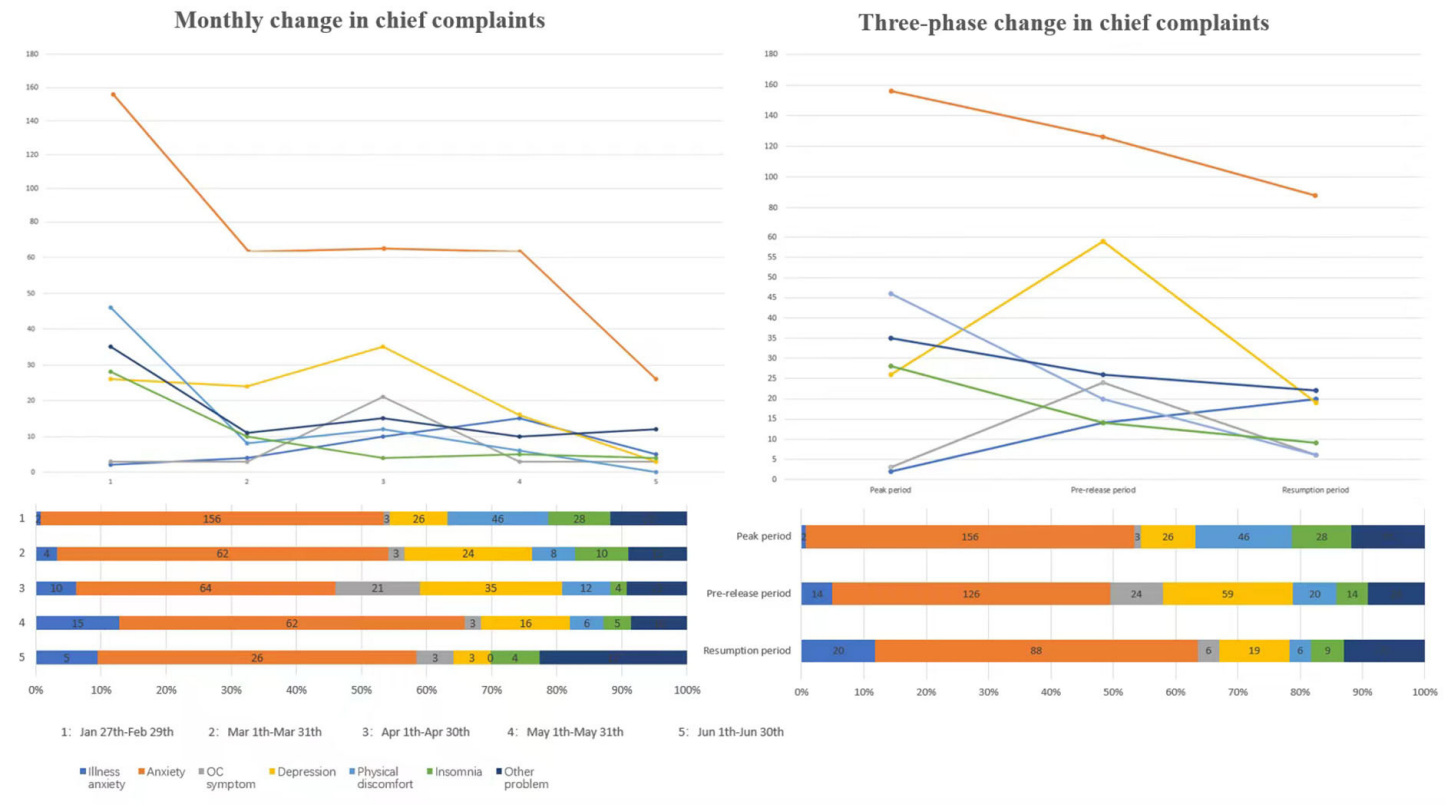
The monthly and three-phase changes in chief complaints of the psychological hotline from 27 January to 30 June 2020. Anxiety was the main complaint, and its proportion was significantly higher than that of other complaints. Overall, calls regarding anxiety, insomnia, physical discomfort, and other problems showed a downward trend, and calls regarding anxiety exhibited a plateau period from March to May. In particular, calls regarding illness anxiety increased. In addition, calls regarding depression and obsessive-compulsive symptoms exhibited a small peak in April, which was during the prerelease period. Other problems include consulting about quarantine, registration, medicine purchase, and local policies.

As shown in Table 2, the proportion of callers with anxiety as the chief complaint remained high throughout the epidemic period, and the highest proportion (81.58%) occurred in March; the proportion of callers with physical discomfort and insomnia was only obvious in February and declined during the later period; the proportion of callers with depression and obsessive-compulsive symptoms was low in the early stage and increased significantly in March and April, reaching as high as 35 and 21% in April, and then gradually decreasing in the later period. Generally, a fluctuating trend was shown (Figure 1), and each main complaint peaked at different stages.

**Table 2:** Monthly changes in chief complaints.

		**Total**	**Illness anxiety**	**Anxiety**	**OC symptoms**	**Depression**	**Physical discomfort**	**Insomnia**	**Other problems**
Jan 27^th^-Feb 29^th^	N	281	2	156	3	26	46	28	35
	%		0.71%	55.52%	1.07%	9.25%	15.37%	9.96%	12.46%
Mar 1^st^-Mar 31^th^	N	76	4	62	3	24	8	10	11
	%		5.26%	81.58%	3.95%	31.58%	10.53%	13.16%	14.47%
Apr 1^st^ -Apr 30^th^	N	100	10	64	21	35	12	4	15
	%		10.00%	64.00%	21.00%	35.00%	12.00%	4.00%	15.00%
May 1^st^-May 31^th^	N	82	15	62	3	16	6	5	10
	%		18.29%	75.61%	3.66%	19.51%	7.32%	6.10%	12.20%
Jun 1^st^-Jun 30^th^	N	39	5	26	3	3	0	4	12
	%		12.82%	66.67%	7.69%	7.69%	0.00%	10	30.77%

Other problems include consulting about quarantine, registration, medicine purchase, and local policy.

#### Three-phase trend

As shown in Table 3; Figure 1, the main complaints during the three stages also had distinct features, respectively. During the peak of COVID-19 infection, the number of national newly diagnosed COVID-19 cases increased rapidly. To curb the spread of the epidemic, the government took measures to control the population flow. Correspondingly, anxiety was the main complaint of callers. Most callers were worried about the new coronavirus. Callers with anxiety complaints had more physical discomfort and insomnia symptoms, but some calls were mainly related to consultation on quarantine policies and registration of drugs. The proportion of callers with depression and obsessive-compulsive symptoms was not high during this period. The spread of the epidemic was basically blocked in the middle period, and the number of patients with new coronary pneumonia in hospitals across the country decreased. The proportion of callers with anxiety remained high, while the proportion with depression and compulsion increased significantly; the proportion with illness anxiety gradually increased, and the proportion with physical discomfort and insomnia declined. Compulsions mainly manifested as repeated medical examinations and repeated disinfection and washing for the fear of illness. During the resumption period, epidemic prevention and control entered into normalization, and almost full work resumption occurred. The proportion of callers with anxiety as the main complaint remained high, but the proportion of callers with depression and obsessive-compulsive behavior declined. At the same time, more calls concerned consultation on resumption of work and local epidemic prevention policies, and the proportion of callers with illness anxiety gradually increased. For example, callers with a negative nucleic acid test were still worried about the sequelae of the new coronary pneumonia. At the same time, we also found a statistical difference in the proportion of calls for specific complaints over the phases (Table 4).

**Table 3:** Proportions of chief complaints during the three-phase period.

		**Total**	**Illness anxiety**	**Anxiety**	**OC symptoms**	**Depression**	**Physical discomfort**	**Insomnia**	**Other problems**
Peak period	N	281	2	156	3	26	46	28	35
	%		0.71%	55.52%	1.07%	9.25%	15.37%	9.96%	12.46%
Prerelease period	N	176	14	126	24	59	20	14	26
	%		7.95%	71.59%	13.64%	33.52%	11.36%	7.95%	14.77%
Resumption period	N	121	20	88	6	19	6	9	22
	%		16.53%	72.72%	4.96%	15.75	4.96%	7.44%	18.18

**Table 4:** Proportions of calls for specific complaints differed statistically over the phases.

	**Illness Anxiety +OC symptoms**	**Anxiety**	**Depression**	**Physical discomfort**	**Insomnia**	**Other problems**	**Statistical test**	***p-*value**
Peak period	5 (1.7%)	156 (52.7%)	26 (8.8%)	46 (15.5%)	28 (9.5%)	35 (11.8%)		
Prerelease period	38 (13.4%)	126 (44.5%)	59 (20.8%)	20 (7.1%)	14 (4.9%)	26 (9.2%)	χ^2^ = 74.94	<0.001
Resumption period	26 (15.3%)	88 (51.8%)	19 (11.2%)	6 (3.5%)	9 (5.3%)	22 (12.9%)		

## Discussion

According to the dynamic observation results and qualitative analysis of the information extracted from the detailed records of the psychological crisis hotline in this study, we found that the proportion of male callers was slightly higher than that of female callers during the COVID-19 outbreak period. However, studies have found that women are more likely than men to develop psychological stress responses, which may be related to their own susceptibility factors, including physical, psychological, and social factors (5, 6), but it is possible that women are more resilient than men. During the COVID-19 outbreak, we applied Zung's Self-Rating Anxiety Scale, Zung's Self-Rating Depression Scale, the Connor–Davidson resilience scale, and Simplified Coping Style Questionnaire to 3,180 people and finally found that individuals with a higher level of mental resilience and active coping styles had a lower level of anxiety and depression (7). In addition, hotline callers were mainly young and middle-aged people aged 19–45 years, which may be related to young people being more receptive to online smart services. To date, several online psychological self-help intervention systems have been developed for online psychoeducation and psychotherapy, including cognitive behavioral therapy or supportive therapy for depression, anxiety, and insomnia. Several artificial intelligence techniques and applications have also been put into use as interventions for psychological crises during the epidemic (8). Meanwhile, publicity efforts should be increased to raise public awareness and make it easier for the public to get professional help themselves.

According to the overall trend, we found that calls regarding anxiety, insomnia, physical discomfort, and other problems showed a downward trend in the monthly and three-phase analysis, mainly in the early stage. The main manifestations of anxiety were nervousness, worry, and fear of contracting or getting infected with the new coronavirus, accompanied by dizziness, palpitation, cough, and other physical symptoms, as well as insomnia. This is a common early psychological response under a stressful epidemic state, and the fear of the uncertainty may also be present. Psychological stress is an interactive dynamic balance “system” composed of an individual's life events, cognitive evaluation, coping style, social support, personality characteristics, and psychosomatic response. When the system is out of balance under the stressful conditions, the individual will experience a state of psychological stress. Moderate psychological stress may motivate individuals to actively face catastrophic events, but excessive psychological stress response may cause individuals to experience hypersensitivity, difficulty concentrating, impaired memory, decreased judgment ability, anxiety and depression, panic and irrational behaviors, and even serious psychological problems such as post-traumatic stress disorder (PTSD). One study conducted five follow-up visits of SARS survivors over a period of 4 years and found that the prevalence of PTSD exceeded 40% (9). In addition, studies have shown that in the early stages of an outbreak lack of information from official channels, misleading information from social media, and fear of the uncertainty can also increase public anxiety (10). According to other hotline reports, during the peak of the epidemic, the “Xinxinyu” hotline of Wuhan Mental Health Center received a total of 2,653 calls from 4 February to 24 February. Among them, 33.5% of callers reported anxiety, 10.1% expressed depressive symptoms, and 5.5% had sleep problems (11). In addition, the crisis hotline of the Brain Hospital affiliated with Guangzhou Medical University received 1,973 calls related to the COVID-19 epidemic from 23 January to 26 March. A total of 523 (26.5%) calls concerned emotional problems directly caused by anxiety, fear, worry, and hypochondriasis symptoms (5, 6). This is also consistent with our observations. We found that complaints of anxiety and physical discomfort accounted for a high proportion of hotline calls during the peak of the epidemic, followed by depression (12). Therefore, psychological crisis intervention in the early stage of the new coronary pneumonia epidemic may have effectively reduced or alleviated the occurrence of related psychological problems or mental disorders. Regarding the early response, psychological intervention mainly focused on normalizing the anxiety response, mainly using core listening skills and providing support to soothe the panic caused by the caller's psychological imbalance, guiding them to normalize and accept the anxiety response, and understand the progress of prevention and control of the epidemic.

In particular, one interesting point was that calls regarding illness anxiety showed a modest upward trend. This may have occurred because the public was overly nervous about information related to the epidemic. If the epidemic is not thoroughly controlled, it will fluctuate periodically. Illness anxiety can be defined as a constant, excessive, and irrational worry that is present despite an absence of physical or psychological disease. COVID-19 causes anxiety because it affects people's lives negatively and brings many uncertainties to society. Since the virus has a high rate of spreading from person to person, it causes pressure in personal relationships, and the anxiety increases due to uncertainties regarding how long the pandemic will last and how long its effects will continue. This is also consistent with our results. Previous studies have found that female gender, accompanying chronic disease, and previous psychiatric history were found as risk factors for illness anxiety (13). In subsequent studies, we can further verify and carry out early prevention for high-risk groups.

We also found that calls regarding depression and obsessive-compulsive symptoms showed a small spike in April. As the quarantine period becomes longer, public mental health will worsen. Individuals with any physical symptoms that may be related to the infection will become repeatedly worried, and some obvious compulsive behaviors will be shown; additionally, people will be quarantined for a long time. Prolonged quarantine often leads to feelings of boredom, depression, and isolation, resulting in significantly higher post-traumatic stress and depression symptoms (14). Moreover, long-term quarantine at home without income causes great economic pressure for families. During this period, psychological intervention should mainly involve providing various types of psychological support. Although population movement is prohibited during an epidemic, this does not mean self-isolation. The public can be encouraged to use mobile phones and the internet to communicate with family and friends and not let themselves fall into a state of self-isolation. If effective adjustments cannot be made for a long time, the public should be advised to seek professional psychological assistance. Second, the most important psychological intervention during this period should be the popularization of psychological science, to inform the public of self-help methods for psychological crises, combined with internet services and smartphones, so that the public can find a sense of self-control, reduce their anxiety level, and increase their sense of security (15). There may be additional social support for people who are isolated, people with low family incomes, and people who have lost income due to the epidemic (14). The study also found that the proportion of callers who consulted about drug purchase, registration, resumption of work in other places, and local epidemic prevention policies was also relatively high, accounting for a considerable proportion during each period. This is mainly because prolonged quarantine will cause difficulties in purchasing drugs and medical treatment, financial difficulties due to lack of work, and lack of knowledge about official policies. Therefore, it is necessary to explore the psychological hotlines (16) and online medical treatment models of internet hospitals (17). To help prevent and alleviate the psychological distress caused by the epidemic, the National Health Commission has launched a national psychological assistance hotline inquiry service, and crisis hotlines across the country will successively provide 24-h free psychological services (18).

In short, based on the guiding principles of psychological crisis intervention during the COVID-19 epidemic, active prevention should be performed while slowing down and trying to control the psychosocial impact caused by the epidemic according to the different psychological characteristics of the public during different periods. Additionally, healthcare professionals should be alert to the various psychological effects and mental disorders after the epidemic, such as post-traumatic stress, persistent depression, anxiety and somatization reactions, persistent obsessive-compulsive disorder, and insomnia. In fact, the number of hotline consultations has increased rapidly in the wake of the COVID-19 outbreak, which may be due to the use of new media to promote mental health knowledge and improve the public's psychological preparation for the crisis. However, the public's demand for psychological services has increased the professional requirements for hotline operators, who need to have corresponding professional qualifications and rich experience in helping others. Therefore, efforts should be made in the following aspects: (1) establish a talent pool; usually, the psychological hotline operators have basic psychological knowledge and basic counseling skills; (2) improve the supervision and training system for professionals; and (3) form clear management system and service standard for the operation of psychological hotline. When other public health crises occur, work on the psychological crisis intervention hotline by different groups can be targeted and adapted as appropriate (19). In addition, in the next step, the caller's psychological changes and mental state can be tracked after the epidemic, and the impact of crisis events on the public can be explored to identify positive response measures.

There are some limitations to this study. The dynamic observation of psychological hotlines combined with qualitative and quantitative analysis can help understand the public's psychological state in a timely manner during an epidemic. The disadvantage of the study is that the sample size is limited, and the study is restricted by the region, with certain regional characteristics. In the future, we can expand publicity and unite with various provinces and even psychological hotline centers across the country to perform this work. In addition, approximately a quarter of the callers had a detailed history of illness, mainly including some common mental disorders and chronic physical diseases. Most of the drugs being taken are psychotropic. We cannot differentiate between the worsening of a preexisting condition and a specific psychological response to the epidemic. Most of the people seeking help were middle-aged and young people who were willing to actively seek help. They can only represent a small number of people, not the whole. Therefore, to fully understand the public's psychological state, the data should be supplemented in other ways.

## Data availability statement

The original contributions presented in the study are included in the article/supplementary material, further inquiries can be directed to the corresponding author/s.

## Ethics statement

The studies involving human participants were reviewed and approved by the Institutional Review Board of the affiliated Nanjing Brain Hospital of Nanjing Medical University. Written informed consent to participate in this study was provided by the participants' legal guardian/next of kin.

## Author contributions

MO and SS contributed to the writing of this article and the statistical analysis of this article. NL led the whole study, including putting forward this study, and carrying out the study. NZ contributed suggestions on revision after review of the manuscript. JLe and HY contributed to the preparation of the psychological crisis hotline. HM, PZ, and CT collected data. HO and JLi contributed to quality control. All authors contributed to the article and approved the submitted version.

## Funding

This study was supported by the National Natural Science Foundation of China (81901390).

## Conflict of interest

The authors declare that the research was conducted in the absence of any commercial or financial relationships that could be construed as a potential conflict of interest.

## Publisher's note

All claims expressed in this article are solely those of the authors and do not necessarily represent those of their affiliated organizations, or those of the publisher, the editors and the reviewers. Any product that may be evaluated in this article, or claim that may be made by its manufacturer, is not guaranteed or endorsed by the publisher.
